# The α-Proteobacteria *Wolbachia pipientis* Protein Disulfide Machinery Has a Regulatory Mechanism Absent in *γ-Proteobacteria*

**DOI:** 10.1371/journal.pone.0081440

**Published:** 2013-11-25

**Authors:** Patricia M. Walden, Maria A. Halili, Julia K. Archbold, Fredrik Lindahl, David P. Fairlie, Kenji Inaba, Jennifer L. Martin

**Affiliations:** 1 The University of Queensland, Institute for Molecular Bioscience, Division of Chemistry and Structural Biology, Brisbane, Queensland, Australia; 2 Division of Protein Chemistry, Medical Institute of Bioregulation, Kyushu University, Higashi-ku, Fukuoka, Japan; University of Cantebury, New Zealand

## Abstract

The α-proteobacterium *Wolbachia pipientis* infects more than 65% of insect species worldwide and manipulates the host reproductive machinery to enable its own survival. It can live in mutualistic relationships with hosts that cause human disease, including mosquitoes that carry the Dengue virus. Like many other bacteria, *Wolbachia* contains disulfide bond forming (Dsb) proteins that introduce disulfide bonds into secreted effector proteins. The genome of the *Wolbachia* strain *w*Mel encodes two DsbA-like proteins sharing just 21% sequence identity to each other, α-DsbA1 and α-DsbA2, and an integral membrane protein, α-DsbB. α-DsbA1 and α-DsbA2 both have a Cys-X-X-Cys active site that, by analogy with *Escherichia coli* DsbA, would need to be oxidized to the disulfide form to serve as a disulfide bond donor toward substrate proteins. Here we show that the integral membrane protein α-DsbB oxidizes α-DsbA1, but not α-DsbA2. The interaction between α-DsbA1 and α-DsbB is very specific, involving four essential cysteines located in the two periplasmic loops of α-DsbB. In the electron flow cascade, oxidation of α-DsbA1 by α-DsbB is initiated by an oxidizing quinone cofactor that interacts with the cysteine pair in the first periplasmic loop. Oxidizing power is transferred to the second cysteine pair, which directly interacts with α-DsbA1. This reaction is inhibited by a non-catalytic disulfide present in α-DsbA1, conserved in other α-proteobacterial DsbAs but not in γ-proteobacterial DsbAs. This is the first characterization of the integral membrane protein α-DsbB from *Wolbachia* and reveals that the non-catalytic cysteines of α-DsbA1 regulate the redox relay system in cooperation with α-DsbB.

## Introduction

*Wolbachia pipientis* is one of the most common bacteria on earth, infecting a wide range of invertebrates, including more than 65% of insect species [1]. *Wolbachia* infects the gonads of its host, altering its reproduction in a variety of unusual ways. In 1971, Yen & Barr first discovered that the cross between healthy female mosquitoes with *Wolbachia*-infected males did not produce viable offspring, a phenotype called cytoplasmic incompatibility (CI) [2]. CI has been described in a wide range of insect species, and has been particularly studied in *Drosophila* [3], [4]. Although CI is the most common form of reproductive manipulation induced by *Wolbachia* in insects, *Wolbachia* can also cause feminization of genetic males, parthenogenesis and male killing, depending on the host species and the *Wolbachia* strain (for review see [4]). All of these phenotypes favor an increased proportion of females in the population, which supports *Wolbachia's* maternal transmission [5]. The ability of some *Wolbachia* strains to inhibit several insect borne diseases such as Dengue viruses, together with the capacity to invade field populations as a consequence of CI induction, has prompted the use of *Wolbachia*-infected *Aedes aegypti* mosquitoes as a novel biocontrol tool against this disease [6]–[11].

The molecular mechanisms that underpin these *Wolbachia*-induced phenotypes remain unclear, but it has been suggested that the bacterium modifies host phenotypes through the action of proteins secreted into the host cytoplasm [12], [13]. A common bacterial secretion pathway is the type IV secretion system (T4SS), which enables bacteria to transfer DNA and proteins into its host [14]. In several *Wolbachia* strains, two conserved operons of the T4SS, virB3-B6 and virB8-D4, have been reported [15]. Although the nature of the molecules secreted into the host environment remains unknown, in close relatives of *Wolbachia* the T4SS is known to secrete effector proteins such as Ankyrin-repeat-containing proteins (ANK) [16]–[19]. Proteins containing ANK domains are often involved in protein:protein interactions, have been shown to connect symbiont cell membranes to the cytoskeleton, and are required for infection of the host cell [16]–[19]. *Wolbachia* genomes are unusual among bacteria in that they contain particularly high numbers of ANK genes [20]–[22]. The high proportion of ANK genes, and the presence of a complete T4SS, suggests that *Wolbachia* secretes effector proteins, such as ANK into its host.

In bacteria, disulfide bond formation is critical for correct folding and stability of secreted effector proteins. The introduction of disulfide bonds is known as oxidative protein folding and requires thiol-disulfide exchange reactions [23]. The disulfide bond forming (Dsb) enzymes of the *Escherichia coli* K-12 strain are known to fold many secreted protein substrates, including toxins and surface proteins that contribute to bacterial virulence [24]. Therefore, it is likely that *Wolbachia* Dsb proteins also play essential roles in maturation of effector proteins, before they are secreted into the host.

In *E. coli*, the oxidative folding pathway requires two proteins, EcDsbA and EcDsbB, that act together to introduce disulfide bonds into newly synthesized proteins [25], [26]. When EcDsbA accomplishes this task, its active site cysteines become reduced and must be re-oxidized to react with another substrate protein. Oxidation of EcDsbA is catalyzed by an integral membrane protein, EcDsbB [27], [28]. EcDsbB contains four transmembrane (TM) helices, two periplasmic loops (P1 and P2), and cytoplasmic N and C-termini. The redox function of EcDsbB requires a pair of cysteines in each of the P1 and P2 loops [27] and its oxidizing power is sourced from a bound cofactor ubiquinone (UQ, redox potential +110 mV [29]), though it is the P2 cysteine pair that interacts directly with EcDsbA [30].

The genome of *Wolbachia* wMel strain encodes two DsbA-like proteins, α-DsbA1 and α-DsbA2 [31], and a predicted integral membrane protein α-DsbB with low sequence identity to EcDsbB. Previous characterization of α-DsbA1 revealed striking differences in the structure of α-DsbA1 compared to that of EcDsbA [31]. α-DsbA1 possesses a second disulfide, that is likely to have a regulatory role since it is not redox-active but it introduces local strain into the enzyme structure [31]. The two cysteines forming the second disulfide are highly conserved in alpha-proteobacterial DsbAs but not in other DsbAs [31]. In addition, the α-DsbA1 structure lacks the characteristic hydrophobic surface features surrounding the active site disulfide of EcDsbA [31]. Finally, α-DsbA1 does not interact with EcDsbB, the redox partner of EcDsbA [31].

Much attention is paid to the *Wolbachia* Dsb proteins because of their potential role in folding *Wolbachia* effector proteins and the interest in using *Wolbachia* as a biocontrol weapon. Here, we investigated whether the predicted *Wolbachia* membrane protein α-DsbB acts as a redox partner of either α-DsbA1 or α-DsbA2. We found that α-DsbB and α-DsbA1 form a functional redox pair and that their interaction mechanism resembles that of EcDsbA:EcDsbB, though the redox pairs are organism-specific: α-DsbA1 does not interact with EcDsbB and α-DsbB does not interact with EcDsbA. Curiously, we found no interaction between α-DsbB and α-DsbA2, suggesting that α-DsbA2 has a distinct role from that of other bacterial DsbAs.

## Materials and Methods

### Production of α-DsbA1, α-DsbA1CA and α-DsbA2

α-DsbA1 and its mutant form, α-DsbA1CA, were expressed and purified as described previously [31], [32]. Full-length α-DsbA2 (locus WD1312; GenBank #AE017196) was amplified from *w*Mel genomic DNA by PCR using the following primers: Forward 5′ TAC TTC CAA TCC AAT GCG ATG AGC TTG CCG ATA ATA 3′ and Reverse 5′ TTA TCC ACT TCC AAT G CT AGC CTT GCT TGT GAC TTA A 3′ to incorporate overhangs for ligation independent cloning. Truncated α-DsbA2Δ50 was amplified using the primers: Forward 5′ TAC TTC CAA TCC AAT GCG GCT CGA GAT AAT GTA ACC 3′ and Reverse 5′ TTA TCC ACT TCC AAT GCT AGC CTT GCT TGT GAC TTA A 3′. The full-length mature protein and α-DsbA2Δ50 were cloned into the LIC vector pMCSG7 that incorporates a HIS_6_-tag, a linker region containing 8 amino acids, and a TEV-cleavage site at the N-terminus of the inserted gene. The construct was transformed into the expression strain BL21(DE3)pLysS (Life Technologies, USA) to enable over-expression of α-DsbA2 using the autoinduction method [33]. Cells were collected using an Avanti J-25I centrifuge (Beckman Coulter, Australia) at 12,000 x g and 4°C for 10 min and frozen at -80°C. α-DsbA2 was expressed and purified as described in [32] with minor variations to the lysis, wash and elution buffers. These buffers contained 25 mM Tris-HCl pH 7.5 (Sigma Aldrich, USA), 150 mM NaCl with 25 mM imidazole for washing, and 250 mM imidazole for eluting the protein.

### Cloning, Expression and Purification of wt α-DsbB

Full-length α-DsbB was amplified from *w*Mel (locus WD1099, Accession No 966820) by PCR using specific primers: Forward 5′ CAT ATG GTC AAT TAT CCC TGA AAG TAT AAA GCA GAG TAT TGC TAG G 3′ and Reverse 5′ CTC GAG TTT AGA TTT CTT TCT TTC ACA ATA CAC 3′ including restriction sites for NdeI and XhoI (underlined). The pET21a vector into which the open reading frame of α-DsbB was inserted was transformed into TOP10 competent cells (Life Technologies, USA) for sequencing.

BL21(DE3)pLysS cells harboring the expression vector of α-DsbB were cultivated in LB media containing ampicillin and chloramphenicol and grown at 30°C until the OD_600_ reached 0.5. Expression of α-DsbB was induced with IPTG (Thermo scientific, USA)(250 µM final) and cells were incubated at 30°C for another 4 h. Cells were harvested in an Avanti J-26 Xpi centrifuge (Beckmann Coulter, USA) at 7, 000 x g for 10 min. Bacterial pellets were resuspended in buffer (10 mL of buffer per 1 g of cells) containing 50 mM NaPO_4_ pH 8.0 (Biochemicals, Australia), 300 mM NaCl (Amresco, USA), 10% glycerol (Chem-Supply, USA), 100 U DNAse (Roche, Australia), and 100 U protease inhibitor cocktail (Roche, USA). Cell disruption was then carried out using the bench top one-shot model (Constant Systems Ltd., USA) at 25 kPsi with two cycles of cell breakage. Unbroken cells and debris were removed by centrifugation at 17, 000 x g for 20 min in an Avanti J-26 Xpi centrifuge (Beckmann Coulter, USA). Membranes were harvested from the supernatant by ultracentrifugation in an Optima L 100 XP (Beckmann, USA) at 100, 000 x g for 1 h at 4°C. The membrane pellet was solubilized in buffer containing 50 mM NaPO_4_ pH 8.0, 300 mM NaCl, 10% glycerol and 1% n-dodecyl-β-D-maltoside (β-DDM) (Affymetrix, USA) for 1 h at 4°C. Solubilized membranes were ultracentrifuged at 100,000 x g for 20 min at 4°C to remove the unsolubilized membrane fraction. The supernatant was then added to TALON® resin (Clontech, Australia) (3 mL TALON® per 50 mL lysate) and incubated for 1 h on a rotation wheel at 4°C. The resin was then washed with buffer as described above and 20 mM imidazole and eluted from TALON® using buffer including 50 mM NaPO_4_ pH 8.0, 300 mM NaCl, 0.03% β-DDM and 250 mM imidazole. Eluted and concentrated protein was injected onto a Superdex™ 200 GL 10/300 column (GE Healthcare, USA) on an ÄKTA FPLC chromatographic system (GE Healthcare, USA). To identify protein-containing fractions, 15 µL samples from peak fractions were applied to a 4–12% Bis-Tris SDS PAGE (Life Technologies, USA). Protein-containing fractions were concentrated in a 50, 000 Da cut-off AMICON concentrator tube (Merck, USA) at 3, 000 x g at 4°C for biochemical assays.

### Preparation of α-DsbB variants [CCSS] and [SSCC]

To create α-DsbB [CCSS] and α-DsbB [SSCC] variants, we used the following primers: Forward 5′- CAT GAT GTT TTA GGT AGT ACA GAG CAA GCA AGT AGT AAC G - 3′ and Reverse 5′ - C GTT ACT ACT TGC TTG CTC TGT ACT ACC TAA AAC ATC ATG 3′, Forward 5′ TAC TCT CCA TCT TCG GAC AGA CCT CAT TAC - 3′ and Reverse 5′ GTA ATG AGG TCT GTC CGA AGA TGG AGA GTA 3′, and Forward 5′ - ATG CTG CCA TCC AAG TTA TCT ACA TAC GAG CGA - 3′ and Reverse 5′ - TCG CTC GTA TGT AGA TAA CTT GGA TGG CAG CAT - 3′, respectively. PCR products were cleaned using the QIAquick Gel Extraction Kit (Qiagen, Netherlands) and transformed into TOP10 cells for sequencing. The expression and purification steps were performed as described for α-DsbB above.

### Circular dichroism (CD) spectra of wt α-DsbB, α-DsbB [CCSS] and α-DsbB [SSCC]

CD experiments were performed with wt α-DsbB, α-DsbB [CCSS], and α-DsbB [SSCC]. Samples were measured in a UV-cuvette (pathlength 1mm) in the far UV region using a J-810 spectropolarimeter (Jasco, USA). Samples had a protein concentration of 5 µM and the buffer (500 µL) contained 50 mM NaPO_4_ pH 8.0, 100 mM NaCl and 0.1% β-DDM. Measurements were performed in duplicate.

### Reduction and oxidation of α-DsbA1 and α-DsbA2

α-DsbA1 and α-DsbA2 were reduced with 20 mM diothiothreitol (DTT reduced) (A.G. Scientific, USA) or oxidized with 1.7 mM copper(II)-1,10-phenanthroline made from a 1∶3 molar ratio solution of CuCl_2_ (Sigma-Aldrich, USA) dissolved in water and 1,10-phenanthroline (Sigma-Aldrich, USA) dissolved in ethanol. The samples were incubated for 30 min on ice. To remove DTT or copper(II)-1,10-phenanthroline, the sample was applied to a PD10-Sephadex column (GE Healthcare, USA) and eluted in degassed buffer containing 50 mM NaPO_4_ pH 8.0, 300 mM NaCl and 0.1% β-DDM. The Ellman assay was performed to verify that the proteins were fully reduced. In this assay, a colorimetric reaction occurs between Ellman's reagent 5,5-dithiobis-2-nitrobenzic acid (DTNB) and free thiol groups. The reagent is used to quantify free thiols in a sample. After reaction of DTNB with a free thiol, TNB^2-^ is released imparting a yellow colour to the solution. The amount of TNB^2-^ (and therefore of free thiol) is quantified by measuring the absorbance of the sample at 412 nm. For these experiments, a 1 ml reaction volume was used with 2 mM of DTNB, and 36.2 µM of the sample protein (α-DsbA1, α-DsbA1CA or α-DsbA2) in Ellman's buffer (80 mM NaPO_4_, 1 mM EDTA, 2% w/v SDS). Under these conditions, an absorbance reading of 1.0 at 412 nm indicates the presence of one free thiol in the protein sample. For example, purified reduced α-DsbA2 gave an OD_412 nm_ reading of 2.05±0.03 (indicating the presence of two free thiols) whereas purified oxidized α-DsbA2 gave an OD_412 nm_ reading of 0.05±0.01 (indicating no free thiols). OD_412 nm_ measurements were made using a CARY 50 Bio UV-spectrophotometer (Varian, USA).

### Ubiquinone reduction assay

The ability of α-DsbB to oxidize reduced α-DsbA1 and α-DsbA2 in the presence of ubiquinone (UQ1, [34]) was assayed spectrophotometrically using a CARY 50 UV-visible spectrophotometer (Varian, USA). The reduction of UQ1 can be measured as a decrease in absorbance at 275 nm. The assay was performed at 30°C in buffer (100 µL) containing 50 mM NaPO_4_ pH 8.0, 100 mM NaCl, and 0.1% β-DDM. α-DsbA1 (30 µM, 40 µL of a 75 µM stock solution) was mixed thoroughly with α-DsbB (50 nM, 5 µL of a 1 µM stock solution) and UQ1 (30 µM, 3 µL of a 1000 µM stock solution). The reduction rates of UQ1 were calculated (using PRISM Version 6.0a [35]) from the decrease in the linear absorbance curve. Measurements were performed in triplicate.

### Redox state analysis of α-DsbA1 and α-DsbA2 in the presence of α-DsbB and variants

Reduced α-DsbA1 (30 µM, 40 µL of a 75 µM stock solution) was mixed with α-DsbB (50 nM, 5 µL of a 1 µM stock solution) and UQ1 (30 µM, 3 µL of a 1000 µM stock solution) in buffer (100 µL) containing 50 mM NaPO_4_ pH 8.0, 100 mM NaCl, and 0.1% β-DDM and 5 µL samples were taken immediately after mixing, and after 0.5 min, 2 min, 5 min, 30 min and 60 min. These samples were mixed immediately with trichloroacetic acetic acid (TCA, 10 µL of a 10% solution) (Sigma Aldrich, USA), incubated on ice (10 min) and centrifuged (20, 000 x g, 10 min, 4°C). The supernatant was carefully discarded, pellets washed (200 µL 100% cold acetone) and centrifuged (20,000 x g, 10 min). The pellets were air-dried at room temperature for 20 min and resuspended in buffer (10 µL) containing 50 mM Tris pH 7.0 (Amresco, USA), 1% SDS (Amresco, USA)) and 4 mM AMS (Sigma, USA) to label free thiols. Samples were loaded onto a 12% Bis-Tris gel. Three replicates were measured.

### Redox potential of α-DsbB [CCSS] and [SSCC]

α-DsbB [CCSS] (2 µM, 3 µL of a 100 µM stock solution) and α-DsbB [SSCC] (2 µM, 3 µL of a 100 µM stock solution) were each incubated in fully degassed buffer (150 µL) containing 100 mM NaPO_4_ pH 7.0, 1 mM EDTA and 100 mM oxidized DTT (Sigma-Aldrich, USA) and varying concentrations of reduced DTT (2 µM – 5 mM) for 24 h at room temperature. After incubation, the reactions were stopped with TCA (10%, 10 µL) and the precipitated protein pellets collected by centrifugation at 16, 000 x g for 10 min at 4°C. The pellets were washed with 100% cold acetone (200 µL) and dissolved in buffer (10 µL) containing 50 mM Tris-HCl pH 7.0, 1% SDS and 2 mM AMS (Molecular Probes, USA). Separation of reduced and oxidized forms was detected on a 12% SDS Bis-Tris PAGE (Life Technologies, USA). The stained gel was scanned and the band intensity of reduced forms analyzed using ImageJ [36]. The analyzed fractions of the reduced state were plotted against the redox buffers. The equilibrium constant K*_eq_* and the redox potential E^0^' were calculated as described previously [31]. Experimental data were measured in duplicate.

### Peptide oxidation by α-DsbA1

EcDsbB was expressed and purified as described previously [37]. The peptide substrate CQQGFDGTQNSCK, with a 1,4,7,10-tetraazacyclododecane-1,4,7,10-tetraacetic acid (DOTA) group amide-coupled to the N-terminus, and a methylcoumarin amide-coupled to the ε-amino group of the C-terminal lysine, was purchased from AnaSpec, USA. Reconstitution of the peptide substrate solution has been described previously [38].

Assays were run on a Synergy H1 Multimode plate reader (BioTek, USA) with excitation at λ = 340 nm and emission at 615 nm. For time-resolved fluorescence, a 100 µsec delay before reading and 200 µsec reading time was employed. The assay was performed in a white 384-well plate (Perkin Elmer, USA). A solution (25 µL) containing 500 nM α-DsbA1 in 50 mM MES, 50 mM NaCl, 2 mM EDTA, pH 5.5 was added to the wells. The assay was initiated by the addition of peptide (16 µM in 25 µL of 50 mM MES, 50 mM NaCl, 2 mM EDTA, pH 5.5) to each well. The final reaction per well contained α-DsbA1 (500 nM, 5 µL of a 5 µM stock solution), α-DsbB (1.6 µM, 5 µL of a 16 µM stock) and peptide substrate. Three replicates were measured.

## Results

### *Wolbachia* α-DsbB is predicted to have the same topology as *E. coli* DsbB

**Figure 1:**
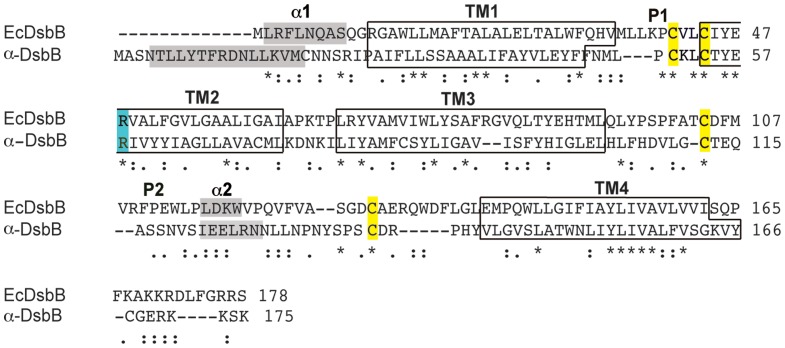
α-DsbB is helical with a predicted topology similar to that of EcDsbB. Sequence-based secondary structure prediction for α-DsbB was derived using *Topcons* [39], *TMHMMfix* [40] and *PsiPred* [42] and the EcDsbB secondary structure was derived from the crystal structure [49]. α-DsbB and EcDsbB both have four conserved cysteine residues (highlighted in yellow), two in each periplasmic loop. The four transmembrane regions of each protein, TM1–TM4, are boxed. Another two helices are present in EcDsbB (α1 at the N-terminus and α2 in loop P2, grey boxes) [56], and these were also predicted in α-DsbB, though the N-terminal helix is much longer than in EcDsbB. Arg48 of EcDsbB and Arg58 of α-DsbB are highlighted in cyan; this residue binds the quinone cofactor in EcDsbB. The sequence alignment of the two proteins was performed using ClustalW [57] with manual adjustments to align loop cysteines and predicted TM helices.

*Wolbachia w*Mel α-DsbB shares just 17% sequence identity with EcDsbB (**Figure 1**). However, a sequence-based structure prediction of α-DsbB suggests that it has the same membrane topology with four TM helices (*Topcons* [39] and *TMHMMfix* [40]). Similar to EcDsbB, α-DsbB has two cysteines located in each of its periplasmic loops: Cys50 and Cys53 in P1 and Cys111 and Cys137 in P2 (**Figure 1**). Moreover, Arg58 of α-DsbB is located at a position equivalent to that of the ubiquinone–interacting residue Arg48 of EcDsbB [**41**] **(** **Figure 1** **)**. α-DsbB is predicted by *Psipred* [42] to contain two short α-helices α1 and α2, where α1 is located at the N-terminus of the protein and α2 in the P2 loop (**Figure 1**). These two helices are also present in the same regions of EcDsbB but both are shorter than the helices predicted in α-DsbB (**Figure 1**). Indeed, the α-DsbB N-terminal region is longer overall than that of EcDsbB (**Figure 1**).

Despite the low sequence identity, α-DsbB and EcDsbB may have a similar function because the predicted membrane topology and the position of critical residues of α-DsbB (*e.g*. the four periplasmic loop cysteines and Arg 58) appear to be conserved.

### CD confirms that α-DsbB is helical

**Figure 2:**
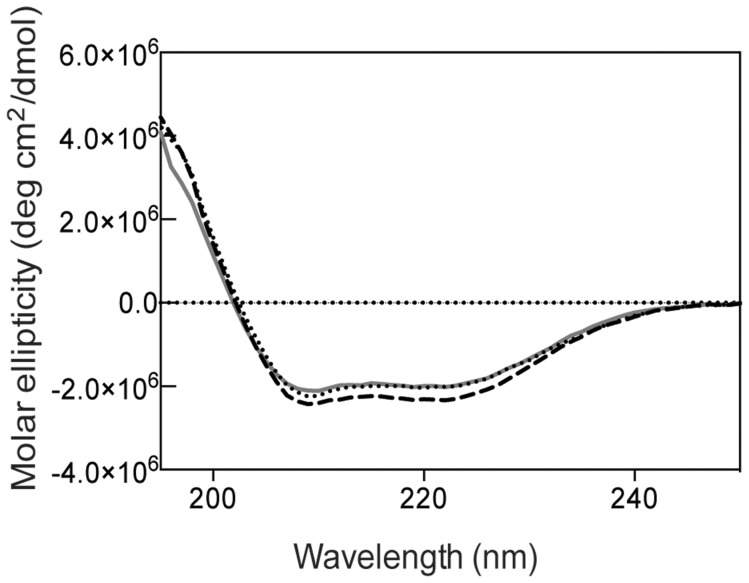
α-DsbB is helical. CD spectra in the far UV region of oxidized wt α-DsbB (dotted line) and [CCSS] (dashed line) and [SSCC] (solid line) variants. The spectra shown are the mean of three replicates.

α-DsbB was produced using recombinant expression methods in *E. coli* and the protein was extracted from membranes by detergent solubilization. The protein-detergent micelles were purified using affinity and size exclusion chromatography, to give a final yield of ∼3 mg of purified α-DsbB from 1L of culture. We also constructed two variants α-DsbB [SSCC] and α-DsbB [CCSS], in which the two cysteines in each of the periplasmic loops were replaced with serines. CD measurement of wild type α-DsbB and both variants revealed far UV CD spectra (**Figure 2**) consistent with a predominantly α-helical protein (84% helix [43]). This finding supports the notion that the overall fold of α-DsbB is similar to that of the helical EcDsbB protein. Furthermore, there was no significant difference between the CD spectra for the wild type and variant forms of α-DsbB, indicating that the overall fold was unaffected by the Cys to Ser mutations (**Figure 2**).

### α-DsbB oxidizes α-DsbA1 but not α-DsbA2

As the role of EcDsbB is to oxidize EcDsbA, the next question to address was whether α-DsbB formed a functional redox pair with either or both α-DsbA1 and α-DsbA2. α-DsbA1 and α-DsbA2 have 21% sequence identity to each other, and 11% and 19% with EcDsbA, respectively (PDB codes: 3F4R, 1DSB) [31], [44]. The structure of α-DsbA1 shows that it has a DsbA fold comprising a thioredoxin domain and an inserted helical domain. The structure of α-DsbA2 is not known though it has a longer N-terminal region than α-DsbA1 and other DsbAs. This region of α-DsbA2 is predicted (PredictProtein [45]) to be helical. A gel-shift assay was used to assess the redox state of both α-DsbA enzymes after incubation with α-DsbB. The α-DsbA enzymes were fully reduced before they were incubated with α-DsbB, and samples were taken from the reaction mixture at a number of time points to investigate the extent of oxidation of the α-DsbA proteins in the presence of α-DsbB and ubiquinone. Detection of the oxidation state was done by labeling free thiols. Thus, thiols of reduced cysteines in α-DsbA1 or α-DsbA2 were labeled with 4-acetamido-4′-maleimidylstilbene-2,2′-disulfonic acid (AMS), which led to an increase in mass (500 Da) for each free cysteine and hence a slower band migration on a 12% Bis-Tris PAGE gel according to the number of free cysteines.

**Figure 3:**
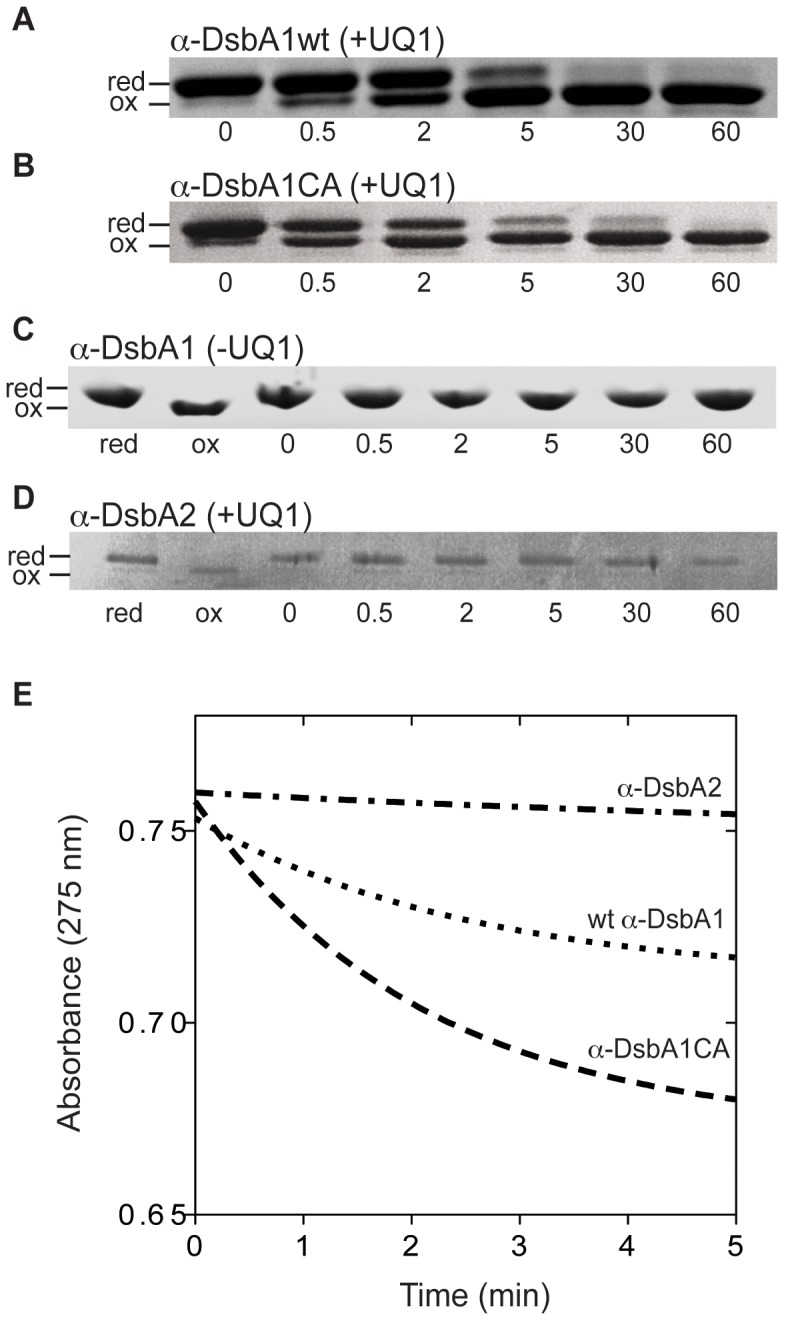
Redox interplay of α-DsbB with α-DsbA1, but not with α-DsbA2. (**A**) α-DsbA1 was oxidized in the presence of α-DsbB and UQ1; (**B**) α-DsbA1CA was oxidized in the presence of α-DsbB and UQ1; (**C**) α-DsbA1 was not oxidized in the presence of α-DsbB and absence of UQ1; lanes marked “red” and “ox” indicate samples of reduced or oxidized α-DsbA1, confirmed by use of Ellman's reagent (**D**) α-DsbA2 was not oxidized in the presence of α-DsbB and UQ1; lanes marked “red” and “ox” indicate samples of reduced or oxidized α-DsbA2, confirmed by use of Ellman's reagent (**E**) Ubiquinone reduction. UQ1 was not reduced when α-DsbB/UQ1 was mixed with α-DsbA2 but was reduced when α-DsbB/UQ1 was mixed with α-DsbA1 or with α-DsbA1CA. Samples of α-DsbA1, α-DsbA1CA and α-DsbA2 were in the fully reduced state (confirmed by use of Ellman's reagent) prior to mixing with α-DsbB/UQ1. The data shown are representative of three replicates.

This assay showed that reduced α-DsbA1 (determined using the Ellman assay) was converted to oxidized α-DsbA1 relatively quickly in the presence of α-DsbB and exogenous ubiquinone-1 (UQ1) (**Figure 3A**). Oxidized α-DsbA1 was detectable within 30 s and oxidation was complete within 10 min. The non-catalytic disulfide of α-DsbA1 has been proposed to play a regulatory role [31], so an α-DsbA1 variant was used in which the cysteines of the non-catalytic disulfide were replaced with alanine (α-DsbA1CA), and this variant was evaluated in the same assay. Interestingly, the time course of oxidation of the variant indicated that it was oxidized more rapidly than wt α-DsbA1. After 30 sec, oxidation appeared to occur 3 times faster for the variant than the wt enzyme and at 2 min and 5 min, respectively, the amount of oxidized variant versus oxidized wt enzyme was 2-fold and 1.3 fold greater. (**Figure 3A, B**). In the absence of exogenous UQ1, α-DsbA1 remained reduced (determined by Ellman assay) over 60 min (**Figure 3C**). The same assay showed that α-DsbB was unable to oxidize α-DsbA2, which remained fully reduced (determined by Ellman assay) throughout the 60 min, even in the presence of exogenous UQ1 (**Figure 3D**). Taken together, the results demonstrated that α-DsbB oxidized α-DsbA1 but not α-DsbA2 and that this reaction required the presence of an oxidizing quinone cofactor.

### UQ is reduced during α-DsbB-catalysed oxidation of α-DsbA1

The observation that α-DsbA1 was oxidized by α-DsbB in the gel-shift assay, and that this oxidation required stoichiometric amounts of exogenous UQ1 suggested that α-DsbB catalysis involved the reduction of UQ and subsequent exchange with oxidized UQ. This is similar to the EcDsbB system [29]. We investigated this possibility by monitoring the decrease of absorbance at 275 nm during the catalytic oxidation of α-DsbA1 by α-DsbB (**Figure 3E**).

When α-DsbB was mixed with α-DsbA2, the exogenous UQ1 was not reduced, confirming the results of the gel shift assay that the two proteins do not form a functional redox pair (**Figure 3E**). However, when α-DsbA1 was mixed with α-DsbB and UQ1, a significant decrease of absorbance at 275 nm was observed, indicating that UQ1 reduction was coupled with α-DsbB-mediated oxidation of α-DsbA1 (**Figure 3E**).

While the initial rate of UQ1 reduction was 0.2361±0.0014 min^−1^ for wt α-DsbA1, the rate increased to 0.4626±0.0016 min^−1^ for the variant α-DsbA1CA (**Figure 3E**). This observation and results from the previous assay (**Figure 3A, B**) suggested that the second disulfide of α-DsbA1 played a regulatory role by inhibiting α-DsbA1 oxidation by α-DsbB.

### Both cysteine pairs in the α-DsbB periplasmic loops are required for α-DsbA1 oxidation

We next investigated the importance of the α-DsbB periplasmic loop cysteine pairs for α-DsbA1 oxidation. In EcDsbB, all four periplasmic loop cysteines are required for oxidation of EcDsbA [46], and we expected that the same would be true for α-DsbB. To test this, the oxidative activity of two variants α-DsbB [CCSS] and α-DsbB [SSCC] was investigated using a gel-shift approach.

**Figure 4:**
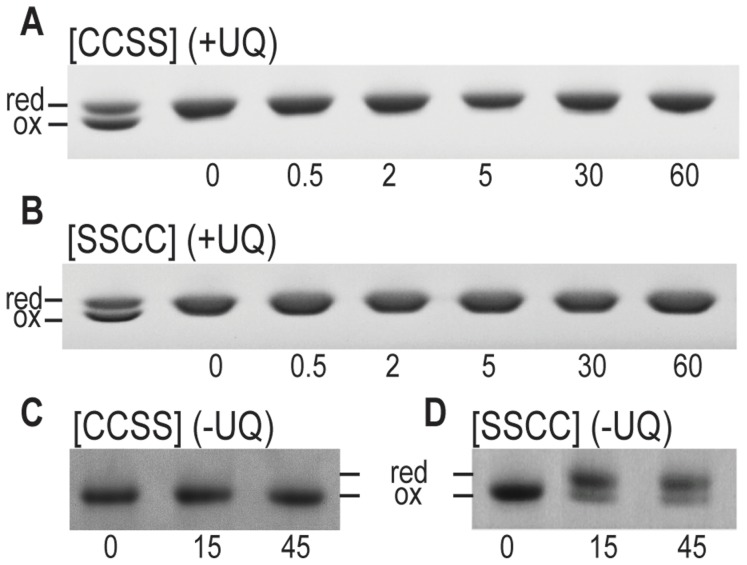
Redox states of wt α-DsbA1 in reaction with α-DsbB [CCSS] and [SSCC]. (**A**) α-DsbA1 was not oxidized in the presence of α-DsbB [CCSS] and UQ1; the first lane shows a reference sample comprising a mixture of reduced and oxidized wt α-DsbA1. (**B**) α-DsbA1 was not oxidized in the presence of α-DsbB [SSCC] and UQ1; the first lane shows a reference sample comprising a mixture of reduced and oxidized wt α-DsbA1. (**C**) Oxidized α-DsbA1 did not oxidize α-DsbB [CCSS] and hence remained oxidized in the presence of α-DsbB [CCSS]; (**D**) Oxidized α-DsbA1 oxidized more than 50% of reduced α-DsbB [SSCC] through thiol-disulfide exchange with reduced α-DsbB [SSCC], yielding significant amount of reduced α-DsbA1. Data shown are representative of two replicates.

The results showed that α-DsbA1 remained fully reduced after 60 min when either of the α-DsbB variants was added in the presence of UQ1 (**Figure 4A and B**), indicating that the cysteine pairs in both periplasmic loops were essential for α-DsbA1 oxidation. In the reverse reaction, we tested whether oxidized α-DsbA1 was able to oxidize the thiols of reduced α-DsbB [CCSS] or reduced α-DsbB [SSCC]. After 45 min, there was no evidence that α-DsbA1 underwent thiol-disulfide exchange with reduced α-DsbB [CCSS] (**Figure 4C**), but more than 50% of α-DsbB [SSCC] was oxidized within 15 min (**Figure 4D**), suggesting that the cysteines in the second periplasmic loop interacted with α-DsbA1.

**Figure 5:**
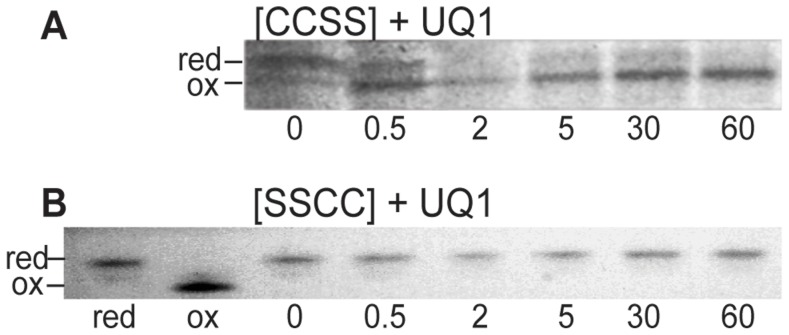
UQ1 interacts with the first periplasmic loop of α-DsbB. (**A**) UQ1 was able to fully oxidize α-DsbB [CCSS] within 2 min; (**B**) UQ1 did not oxidize α-DsbB [SSCC]; lanes marked “red” and “ox” lanes indicate samples of reduced and oxidized α-DsbB [SSCC], respectively. Data shown are representative of two replicates.

The oxidation of reduced α-DsbB [CCSS] and [SSCC] by exogenously added UQ1 was also assessed. UQ1 re-oxidized α-DsbB [CCSS] rapidly, within 2 min (**Figure 5A**). However, UQ1 could not oxidize the α-DsbB [SSCC] variant, which remained reduced over the 60 min incubation (**Figure 5B**). These results suggested that the cysteines of the first periplasmic loop interact with UQ1 and that the cysteines of the second periplasmic loop interact with α-DsbA1, as part of a redox relay.

### Redox potentials of α-DsbB cysteine pairs are more reducing than that of α-DsbA1

EcDsbA has a standard redox potential of −122 mV [47], which is highly oxidizing for thioredoxin superfamily proteins. However, the redox potentials of DsbA enzymes varies considerably: α-DsbA1 is one of the most reducing with a redox potential of −163 mV [31]. We determined the redox potentials of the two essential cysteine pairs in α-DsbB and compared them to those of EcDsbB.

**Figure 6:**
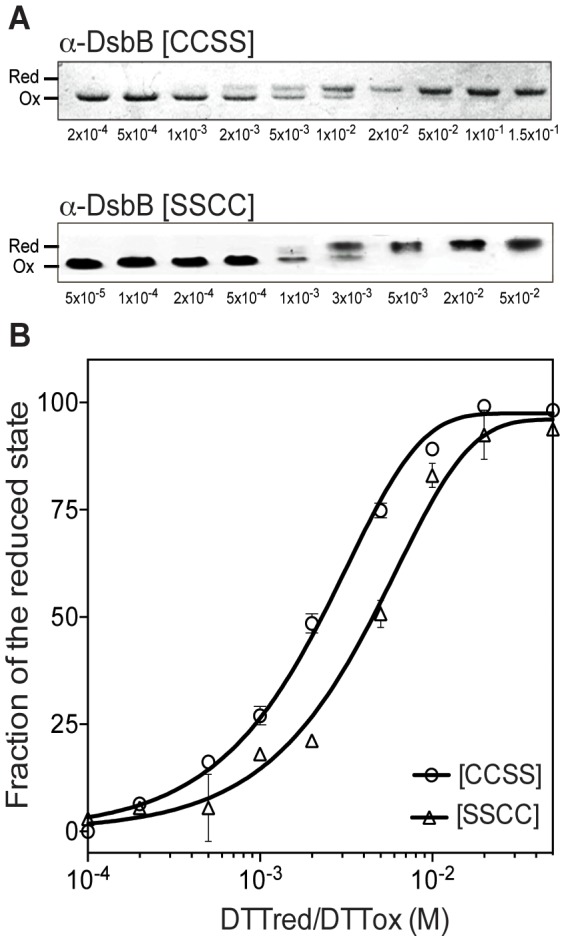
Redox potentials of the cysteine pairs in the P1 and P2 loops of α-DsbB. (**A**) Redox equilibria of α-DsbB [CCSS] and α-DsbB [SSCC]. After equilibration in redox buffers of varying [DTTred]/[DTTox] ratios for 24 h, samples were treated with 10% TCA and free cysteines were labeled with AMS. Reduced and oxidized proteins were separated by 12% SDS-PAGE under non-reducing conditions. (**B**) Redox potentials of α-DsbB [CCSS] and α-DsbB [SSCC]. The fractions of α-DsbB [CCSS] or α-DsbB [SSCC] in the reduced state were calculated using ImageJ [36] and equilibrium constants were plotted against the fraction of the reduced state in PRISM® using the one phase decay option. The data presented are the standard mean and error from duplicate measurements.

We used the α-DsbB variants [CCSS] and [SSCC] to determine the redox potential of the two cysteine pairs by gel-shift analysis of their reduced and oxidized states at various ratios of oxidized DTT (DTT^ox^) versus reduced DTT (DTT^red^) (**Figure 6A**). We found that the redox potentials of the variants [CCSS] and [SSCC] were more reducing than those measured for the counterparts of EcDsbB [46]. Thus, from the experimentally determined equilibrium constants, the redox potentials of αDsbB were calculated to be -251 mV and −275 mV for the cysteine pair in the P1 loop and the P2 loop, respectively (**Figure 6B**). In comparison, EcDsbB has redox potential values of −207 mV for the P1 and −224 mV for the P2 loop cysteine pairs [46]. These results show that in both *E. coli* and *Wolbachia*, the highly oxidizing UQ cofactor (redox potential, +110 mV) is critical to enable DsbB to oxidize DsbA efficiently.

### α-DsbB and α-DsbA1 form a functional redox pair that oxidizes peptide cysteines

Finally, the redox partnership of α-DsbA1 and α-DsbB was probed by assessing their ability to catalyze cysteine oxidation in a peptide substrate. In this assay, a synthetic peptide was used containing two cysteines and tetraazacyclododecane-1,4,7,10-tetraacetic acid (DOTA) and Europium (Eu) doped methylcoumarin groups at the N- and C-termini, respectively. When a disulfide is formed between the two cysteines of the peptide, DOTA and Eu are brought into close proximity, resulting in fluorescence emission. An increase in fluorescence is indicative of disulfide formation in the peptide.

**Figure 7:**
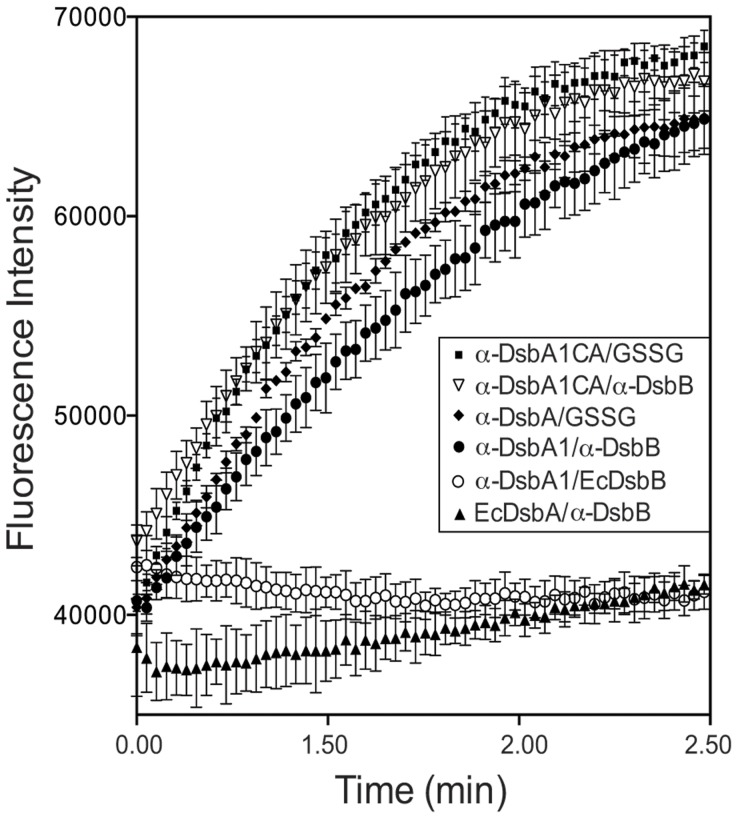
Peptide dithiol oxidation catalyzed by α-DsbA1. Fluorescence curves showing oxidation of a thiol-containing peptide catalyzed by: α-DsbB membranes with α-DsbA1 and α-DsbA1CA; EcDsbB membranes with α-DsbA1; α-DsbB membranes with EcDsbA; or glutathione with α-DsbA1 and α-DsbA1CA. Data represent the standard mean and error of three replicates.

The rate of peptide oxidation by α-DsbA1 was assessed in the presence of three different oxidation sources: (i) α-DsbB (crude membrane containing endogenous UQ); (ii) oxidized glutathione buffer; or, (iii) EcDsbB (crude membrane containing endogenous UQ). For the wild type α-DsbA1/α-DsbB membranes, the peptide was oxidized at an initial rate of 4.77×10^−3^ (±2.9×10^−6^) min^−1^ (**Figure 7**). In the presence of wt α-DsbA1/GSSG, peptide oxidation occurred at a similar initial rate of 4.82×10^−3^ (±2.4×10^−5^) min^−1^. However, with α-DsbA1/EcDsbB membranes no peptide oxidation was observed, indicating that α-DsbA1 did not interact with EcDsbB. Likewise, EcDsbA was incapable of catalyzing peptide oxidation in the presence of α-DsbB membranes.

The activity of the variant α-DsbA1CA was also investigated in this assay, in the presence of wt α-DsbB membranes. Under the same conditions, the peptide was oxidized more rapidly by α-DsbA1CA than by wt α-DsbA1 (6.21×10^−3^ (±3.4×10^−6^) min^−1^).

## Discussion

The introduction of disulfide bonds into bacterial effector proteins is essential for their stability and function. *Wolbachia* encodes two putative periplasmic DsbA-like proteins, α-DsbA1 and α-DsbA2, and a putative integral membrane protein, α-DsbB. Sequence-based structure prediction suggested that α-DsbB has the same overall membrane topology as EcDsbB, and we proposed that the cysteine pairs in the two periplasmic loops (P1 and P2) of α-DsbB would play a similar role to those of EcDsbB in the oxidation of EcDsbA. Indeed, we showed that α-DsbB was a helical membrane protein and that α-DsbB and α-DsbA1 formed a functional redox pair with a highly specific electron flow pathway driven by UQ. The *W. pipientis wMel* genome contains a gene encoding a UQ biosynthesis protein Coq7 (accession code: IP011566), suggesting the biosynthesis of UQ in *Wolbachia* is similar to that in *E. coli* [48].

The α-DsbB variants [CCSS] and [SSCC] were used to measure the redox potentials of the cysteine pairs of P1 and P2 which are both relatively reducing, at −252 mV and −273 mV, respectively. This observation raises the question of how wt α-DsbB can oxidize α-DsbA1 which has a more oxidizing redox potential (−163 mV for wt α-DsbA1 and −161 mV for α-DsbA1CA [31]) than the cysteine pairs of α-DsbB. A similar observation was made for the EcDsbB:EcDsbA system. In EcDsbB, the UQ cofactor is highly oxidizing (redox potential +110 mV), is essential for the oxidation relay and plays a key role in overcoming the unfavourable redox potential difference between EcDsbB and EcDsbA [27]. Moreover, previous studies suggested that three structural elements would be involved in efficient DsbB catalysis of DsbA oxidation: i) DsbA-induced separation of Cys104 from Cys130 in DsbB [25], [49], [50]; (ii) a membrane-parallel α-helix of EcDsbB that is peripherally bound to the membrane and restricts movement of Cys130 [51]; and, (iii) transient formation of an interloop disulfide bond Cys41 and Cys104 in EcDsbB [52], [53]. All three mechanisms likely contribute to preventing the backward attack of liberated EcDsbB Cys130 on the intermolecular disulfide bond formed between EcDsbB Cys104 and EcDsbA Cys30. Since α-DsbA1 lacks the hydrophobic peptide-binding groove of EcDsbA [31], a similar α-DsbA1-induced conformational change of α-DsbB may not be relevant in this system. However, α-DsbB is predicted to have an α-helix in the P2 loop which could play a similar role to the membrane-parallel helix of EcDsbB, and the essential cysteines of α-DsbB are located at positions equivalent to those of EcDsbB (**Figure 1**). The oxidative systems of *E. coli* and *Wolbachia* thus appear to operate in a similar manner to catalyze protein disulfide formation.

On the other hand, the data presented here provides strong evidence that the *E. coli* and *Wolbachia* DSB systems are organism specific. That is, α-DsbA1 oxidized a synthetic peptide when its organism specific redox partner α-DsbB/UQ was present but not when EcDsbB/UQ replaced its partner. Conversely, EcDsbA oxidized a cysteine-containing peptide in the presence of EcDsbB/UQ, but not in the presence of α-DsbB/UQ [54]. This specificity could be attributed to protein surface differences between EcDsbA and α-DsbA1 and sequence differences between the P2 loops of EcDsbB and α-DsbB. The active site disulfide of EcDsbA is adjacent to a hydrophobic groove that interacts with EcDsbB [24], [31], [44] by accommodating the residues PSPFATCDF of the P2 loop (**Figure 1**). There is no equivalent groove in α-DsbA1, and the region around the α-DsbA1 active site contains basic residues [31]. In parallel, the residues of the α-DsbB P2 loop (FHDVLGCTE) are less hydrophobic and more acidic than those of EcDsbB (**Figure 1**).

Another remarkable difference between the two systems is the presence of two cysteines in the helical domain of α-DsbA1 in addition to the redox active site cysteines in the thioredoxin domain. We had noted this second disulfide previously [31], and found that: (i) the two cysteines are highly conserved in α-DsbAs; (ii) the disulfide is not redox-active but introduces local stress into the α-DsbA1 structure; and, (iii) the disulfide does not affect the intrinsic redox properties of α-DsbA1 [31]. The high degree of conservation and the unusual conformation of the second disulfide suggested that it might play a regulatory role. Indeed, oxidation of the two cysteines in the helical domain almost halved the reaction rate of α-DsbA1 with α-DsbB, and the rate of model peptide oxidation by α-DsbA1 was also reduced. This discovery implies that the *Wolbachia* α-DsbA1:α-DsbB oxidative system is subject to feedback regulation through redox-sensing by these cysteines. In this respect, the *Wolbachia* Dsb system may share regulatory features of the eukaryotic endoplasmic reticulum (ER) Ero1p:PDI oxidative system [55] in which ER hyperoxidation is prevented by attenuation of Ero1p activity through noncatalytic cysteine pairs. It will be interesting to determine the redox potential of this putative regulatory disulfide and investigate which molecules regulate its formation/cleavage under physiological condition.

In summary, *Wolbachia* α-DsbB and α-DsbA1 form a functional redox pair analogous, but not equivalent, to that of *E. coli*. By analogy to the *E. coli* system, α-DsbA1 and α-DsbB constitute an oxidative pathway required for the oxidative folding of cysteine-containing effector proteins in the *Wolbachia* periplasm. The two enzymes in the *E. coli* and *Wolbachia* systems are not interchangeable. EcDsbA uses a hydrophobic groove absent in α-DsbA1 to interact with its EcDsbB partner; and *Wolbachia* α-DsbA1 has regulatory non-catalytic cysteines absent in EcDsbA. The role of the second *Wolbachia* DsbA enzyme, α-DsbA2, in oxidative folding requires further investigation, but clearly it does not play the same role as α-DsbA1 or EcDsbA because it does not interact with α-DsbB.
